# A Hybrid Multimodal Emotion Recognition Framework for UX Evaluation Using Generalized Mixture Functions

**DOI:** 10.3390/s23094373

**Published:** 2023-04-28

**Authors:** Muhammad Asif Razzaq, Jamil Hussain, Jaehun Bang, Cam-Hao Hua, Fahad Ahmed Satti, Ubaid Ur Rehman, Hafiz Syed Muhammad Bilal, Seong Tae Kim, Sungyoung Lee

**Affiliations:** 1 Department of Computer Science, Fatima Jinnah Women University, Rawalpindi 46000, Pakistan; asif.razzaq@fjwu.edu.pk; 2 Ubiquitous Computing Lab, Department of Computer Science and Engineering, Kyung Hee University, Seocheon-dong, Giheung-gu, Yongin-si 17104, Republic of Korea; 3 Department of Data Science, Sejong University, Seoul 30019, Republic of Korea; 4 Hanwha Corporation/Momentum, Hanwha Building, 86 Cheonggyecheon-ro, Jung-gu, Seoul 04541, Republic of Korea; 5 Department of Computing, School of Electrical Engineering and Computer Science (SEECS), National University of Sciences and Technology (NUST), Islamabad 44000, Pakistan

**Keywords:** emotion recognition, user experience, audio-based emotion recognition, feature fusioning, decision fusioning, generalized mixture function

## Abstract

Multimodal emotion recognition has gained much traction in the field of affective computing, human–computer interaction (HCI), artificial intelligence (AI), and user experience (UX). There is growing demand to automate analysis of user emotion towards HCI, AI, and UX evaluation applications for providing affective services. Emotions are increasingly being used, obtained through the videos, audio, text or physiological signals. This has led to process emotions from multiple modalities, usually combined through ensemble-based systems with static weights. Due to numerous limitations like missing modality data, inter-class variations, and intra-class similarities, an effective weighting scheme is thus required to improve the aforementioned discrimination between modalities. This article takes into account the importance of difference between multiple modalities and assigns dynamic weights to them by adapting a more efficient combination process with the application of generalized mixture (GM) functions. Therefore, we present a hybrid multimodal emotion recognition (H-MMER) framework using multi-view learning approach for unimodal emotion recognition and introducing multimodal feature fusion level, and decision level fusion using GM functions. In an experimental study, we evaluated the ability of our proposed framework to model a set of four different emotional states (*Happiness*, *Neutral*, *Sadness*, and *Anger*) and found that most of them can be modeled well with significantly high accuracy using GM functions. The experiment shows that the proposed framework can model emotional states with an average accuracy of 98.19% and indicates significant gain in terms of performance in contrast to traditional approaches. The overall evaluation results indicate that we can identify emotional states with high accuracy and increase the robustness of an emotion classification system required for UX measurement.

## 1. Introduction

Over the last decade, the research and applications of multimodal emotion recognition have become increasingly emerging to cater emotional states [1]. Real-time analysis of emotional states through diverse data sources has become one of the most demanding and important research fields [2]. With the appearance of such a diverse technology ecosystem, the concept of multimodality arose very naturally and has brought us numerous breakthroughs for the use of emotions in the fields like affective computing, human–computer interaction (HCI), education, gaming, customer services, healthcare, user experience (UX) evaluation, etc. The application of emotion recognition methods in UX evaluation, however, has not always been so straightforward. As UX has become an essential process to measure the user’s satisfaction and usability, for which emotion can act as a key aspect for evaluating practical applications or software products [3].

Most of the applications adopt human emotion recognition by automatically detecting, processing and performing analysis of human emotions obtained through raw sensory data. The possibility of integrating emotions from multimodal data for UX evaluation to assess the user’s satisfaction and engagement is further reinforced by an overall recognition accuracy and robustness [4]. A possible solution to improve multimodal emotion recognition accuracy is to deal with misclassification defects in some modalities, which may be compensated by some other modality. Finally, obtained emotions from multimodal data might offer helpful feedback for future UX enhancements [5].

Physiological signals analysis, face expression analysis, audio signal analysis, and text input analysis are some of the most frequently used multimodal emotion sensing modalities that might be taken into account while designing a method for UX evaluation. For these, numerous machine learning and deep learning methods have been employed to track features derived independently from each sensory modality and then fuse them either in feature level or decision level [6].

On the other hand, each of the learning method has its own advantages and disadvantages, hence, drawing a general conclusion about the emotions obtained from multi-modalities is challenging. Using different algorithms for each modality usually show inconsistent classification confidence due to the nature of different feature set used, obtained for each modality. From this point of view, it becomes necessary to adopt ensemble-based system for the fusion of output from multiple classifiers [7]. In the literature, various ensemble-based systems have proven themselves to be very effective and have shown their importance in reducing variability, thereby improving the accuracy of automatic human emotion recognition in a multimodal environment. The majority of classifier ensembles apply procedures to define static weights, which are used along with the outputs of the individual classifiers to define the final output of the classifier ensembles. As the accuracy of a single classifier might fluctuate in the testing search space, a static method of generating weights may eventually become inefficient for a classifier ensemble. One way to improve the efficiency is to use the dynamic weights in a combination methods, for this we aim to use a dynamic weighting method supported by generalized mixture (GM) functions [8]. The main advantage of the GM functions is the ability to specify dynamic weights at the member output, which increases the effectiveness of the combination process.

The objective of this article is to enhance the precision of decision fusion. To achieve this goal, we have implemented a novel approach for multimodal fusion in UX evaluation, using GM functions as an efficient combination procedure. Secondly, to improve the UX evaluation process by recognizing and understanding users’ emotions in real-time, we developed a multimodal input collection module that supported cross-modality sensing (CMS) and conducted temporal alignment (TA) of stream events to acquire multimodal data. We evaluated our proposed method on a dataset consisting of individuals’ audio, video, and body language recordings while interacting with stimuli designed to elicit various emotions. Our study revealed that the emotional UX can be enhanced through our proposed approach, which involves real-time detection and understanding of user emotions. We suggested combining data from various modalities through feature-level and decision-level fusion to improve the accuracy of emotion recognition.

The rest of the paper is structured as follows, Section 2 describes related work. Section 3 discusses the proposed approach. Section 4 compares experimental evaluations. Finally, Section 5 draw conclusions and future work.

## 2. Related Work

Much effort has focused on developing frameworks for extracting human emotions from a single modality such as text, video, and audio. However, the robustness of unimodally recognised emotions is still lacking and making it more challenging for multimodal emotions recognition due to inter-modality dependencies. There are various ways in which human emotions have been suggested to be used for different purposes in the literature, such as: emotion recognition, sentiment analysis, event detection, semantic concept detection, image segmentation, human tracking, video classification, and UX enhancement.

UX evaluation, one of the aforementioned approaches, utilizes human emotions, to cover various aspects for the effective use of a product, service, or complete system [9]. The movement of facial muscles, specifically the inner and outer brows, is utilized by humans to deliberately or unintentionally communicate emotional cues. Consequently, a thorough facial expression analysis may effectively identify active muscle groups involve in different emotional responses, such as *Anger*, *Sadness*, *Joy*, *Surprise*, and *Disgust*. So, a deeper understanding of human emotional reactions can be produced through an automatic facial expression analysis. Similarly, speech with different voice characteristics such as intensity, speech rate, pitch, spectral energy distribution, prosodic and acoustic features, also plays an important role to identify human emotions [10].

Non-verbal body gestures, or body language, are equally crucial to emotion recognition as visual and audio-based modalities. They can also provide a critical context for understanding how users engage with the applications mentioned previously. Most of the applications deploy cameras, or depth cameras, to detect emotions by capturing user’s body language. With the increasing number of sensing modalities, the integration of these modalities poses more challenges in multimodal environments. Therefore, a mechanism for multimodal fusion is necessary to process features, make decisions, and perform analysis tasks [11].

Prior studies on multimodal fusion have adopted different research approaches and methods. Among these methods, feature-level fusion (early fusion) and decision-level fusion (late fusion) are the two most common studies that researcher mostly focused on. Ma et al. [12] proposed a cross-modal noise modeling and fusion methodology over multimodal audio and visual data. For this, they trained a 2D convolutional neural network (2D-CNN) model using the image-based mel-spectrograms as input data and a 3D-CNN for detecting emotions from facial expressions in an image sequence. They, however, worked mainly on preprocessing tasks such as handling noisy audio streams and reducing redundancy by proposing time-based data segmentation. Deep convolutional neural network (DCNN) was also utilized for automatic feature learning using discriminant temporal pyramid matching (DTPM) in speech emotion recognition tasks [13].

Li et al. [14] suggested a novel approach to perform multimodal fusion through the utilization of multimodal interactive attention network (MIA-Net). They only considered the modality that had the most impact on the emotion to be the primary modality, with every other modality termed as auxiliary. It may, however lead to a bias towards primary modality and potentially overlook important information from auxiliary modalities. Therefore choosing an appropriate approach for multimodal fusion may lead to certain benefits such as (1) possibility of more accurate predictions; (2) ability to collect information that is not observable in each modality alone; and (3) ability for a multimodal application to continue functioning even if, any of the modalities is absent [15].

A comprehensive review of emotion identification system with underlining basic neural network classification models are described in a study by Gravina et al. [16]. They offered a framework for standard comparison and a methodical classification of the literature on data-level, feature-level, and decision-level multi-sensor fusion approaches. A strategy of utilizing data from vision and inertial sensors for feature-level fusion was also adapted by Ehatisham et al. [17]. They examined and validated the effectiveness of feature-level fusion in contrast with the results obtained from decision-level fusion methods. Radu et al. [6] proposed a modality-specific architecture to demonstrate the capabilities of feature learning to produce accurate emotion recognition results. They demonstrated feature concatenation irrespective of sensing modality and ensemble classification for integrating conflicting information from diverse sources.

As described earlier, each multimodal fusion strategy has its merits and demerits. However, multimodal decision-level fusion overcomes the drawbacks of early fusion techniques to improve the performance of any emotion recognition system. The outcomes of each emotion model are combined for prediction, using various integration techniques including averaging, majority voting, ensemble classification, weighting based on channel noise, signal variance, or through a learned model [18]. Thuseethan et al. [19] have used a hybrid fusion approach to extract and appropriately combine correlated features from face, body pose, and contextual information. Wang et al. [20] designed hybrid fusion model, which combined feature-level fusion and decision-level fusion by finding correlation properties between the features extracted from different modalities. The final emotion state is computed with the help of a combination strategy based on either an equal weights or a variable weight scheme. Przybyla et al. [21] proposed a fusion method for a joint prediction which is provided through a group of classifiers using multiplicative weighting method where weights are assigned iteratively.

The main disadvantage of ensemble-based decision-level fusion is the use of weighting strategies to combine independent and stand-alone classification decisions related to each sensory modality with aim of generating a precise prediction [22]. Therefore, N-classifiers must be trained and evaluated individually on each sensing modality to perform decision-level fusion. There are several methods for computing weights to increase the confidence of each class belonging to a classifier. These decisions are combined by minimizing an error criterion or using weighted voting schemes in ensemble classification [23,24].

In the presented study, to evaluate the performance of emotions, we performed empirical analysis of ensemble classifier using the GM functions combination method for the LeanUX platform [25]. In this method, the output from many independent classifiers is combined using GM functions as a mean of combination. The key benefit of these GM functions is the ability to define dynamic weights at the member classifier outputs, which increases the effectiveness of the combination process. The weight of each classifier is dynamically determined throughout the combination process and does not even require any training. These GM functions generalize dynamic weights based on mixture functions and ordered weighted averaging (OWA).

Overall, the main contribution of this article is fourfold: (1) We propose a hybrid multimodal emotion recognition (H-MMER) framework with multi-level fusion such as unimodal emotion recognition, multimodal feature fusion, and decision-level fusion. (2) An ensembling method within the H-MMER framework utilizing the GM functions has been adopted, which is capable of producing more accurate and consistent emotion recognition predictions. (3) A novel weight combination approach using the GM functions is suggested to assign dynamic weights to each emotion from an individual modality. The proposed framework captures multimodal time-varying data and estimates the joint emotions with high accuracy even without constructing or training an additional model. (4) As a practical contribution, we evaluated our framework in an ongoing research platform called the “LeanUX platform”, which uses acquired emotions for UX measurement, while users interact with the system, product, or a service.

## 3. Proposed Multimodal Emotion Recognition Method

**Figure 1:**
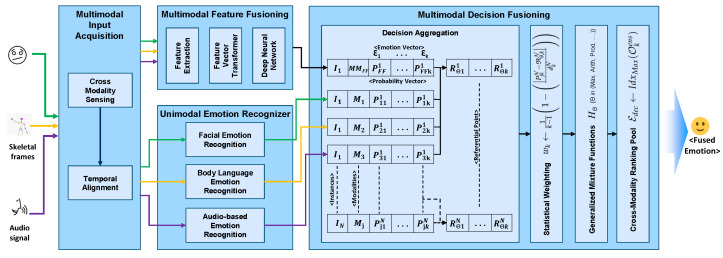
Hybrid Multimodal Emotion Recognition (H-MMER) framework.

We propose a Hybrid Multimodal Emotion Recognition (H-MMER) framework to fuse incoming sensory data streams. The feature that distinguishes multimodal interfaces from unimodal interfaces is the fusion of input modalities. The H-MMER framework extracts emotions from a set of heterogeneous input modalities and passes the fused results to a UX measurement engine within the LeanUX platform [25]. The H-MMER framework comprises several modules executed at three levels: *unimodal emotion recognition*, *multimodal feature fusion*, and *multimodal decision level fusion*, as shown in Figure 1.

The proposed H-MMER framework works on the top of five different types of architectures, which can successively manage an overall emotion score. These architectures include *Unimodal Emotion Recognizers*, *Facial Emotion Recognition*; *Audio-based Emotion Recognition*; *Body Language Emotion Recognition*; and *Multimodal Emotion Recognizers*, such as *Feature Fusion* and *Decision Fusion*. The user’s session log comprises the emotions sensed from multiple modalities for effective UX measurement. Through various experiments, we evaluated and demonstrated analytical results that prove the enhanced emotional representation capabilities of an independent modality or paired modalities when provided with multiple modalities during the feature learning process [26].

### 3.1. Multimodal Input Acquisition

  Most of the multimodal emotion recognition repositories and frameworks are constituted for video or audio modalities. However, a very few of them have considered additional modalities for emotion recognition from text and body language [27]. Through a detailed literature survey, we have identified that there are very few available approaches that integrate *Video*, *Audio*, *Body Movements*, and *text* information. In the development of the H-MMER framework, we developed a *Multimodal Input Acquisition module*, which not only assists *cross-modality sensing* (CMS) but also performs temporal alignment (TA) of stream events for measuring emotions accurately. CMS thus acquires data streams in a real-time from heterogeneous data sources. It provided intelligence to the H-MMER framework based on the sensed modality for activating the unimodal and multimodal emotion recognizers. According to the nature of the modality data, a label is assigned for persistence.

*Unimodal Emotion recognizer* does not require synchronized data from heterogeneous sources. However, *Multimodal Emotion Recognizer* requires time-synchronized closely coupled modalities, where features are extracted from data collected through independent sensors. Synchronization is performed based on the timestamps collected from streams capturing events performed by the users. For this, we also devised the feature concatenation (FC) strategy, which proved to be attractive in terms of simplicity. However, it may miss critical intra-modality correlations. Thus, H-MMER framework ensured strong intra-modality and cross-modality relations supported by deep learning-based modality-specific support.

### 3.2. Uni-Modal Emotion Recognition

In the proposed framework H-MMER, unimodal emotion recognition is a primary building block for individual modality emotion recognition. This section largely focuses on facial, audio, and body language recognizing modalities. The generated output from individual unimodal modules is finally integrated with the feature fusion module before performing decision-level fusion.

#### 3.2.1. Video-Based Emotion Recognition

A webcam was deployed for capturing a stream of frames of size 1024×720 at a rate of 30 fps for human face detection and facial emotion recognition. Prior to analysis, each frame underwent pre-processing, such as cropping, to obtain suitable pixel resolution for the face region, which forms the region of interest (ROI). So, ROI was utilized to speed up other generic object recognition characteristics using a histogram of oriented gradients (HoG) feature descriptor to deal with feature invariances [28]. Moreover, scale-invariant feature transform (SIFT) algorithm was also used to extract and optimize the feature points and remove mismatching results [29]. HoG and SIFT are immune to image transformation techniques such as translation, rotation or scaling, that is why they are used to detect accurate facial features. HoG represents gradient orientation and distribution for ROIs, i.e., a localized part of an individual frame. SIFT computes key points using multi-scale Gaussian filters in the frame termed local features, fully describing the neighborhood features. The output from HoG and SIFT is a combined feature vector representing facial region features. As frames are obtained using a webcam service, it is important to enhance frame quality by applying suitable preprocessing techniques for noise reduction and contrast enhancement. For the former, a non-linear spatial filter method dealing with non-linear local and global information is applied, whereas, for the latter, histogram equalization (HE) is considered to improve contrast for ROIs while preserving key background illumination features.

As discussed earlier, the proposed module extracts key facial expression features and feeds to the input layer of a pre-trained Convolutional Neutral Network (CNN) model in specific ImageNet [30], which maps low-level features to high-level features. Furthermore, layer-wise fine-tuning of the model is performed using baseline video-based facial features.

**Figure 2:**
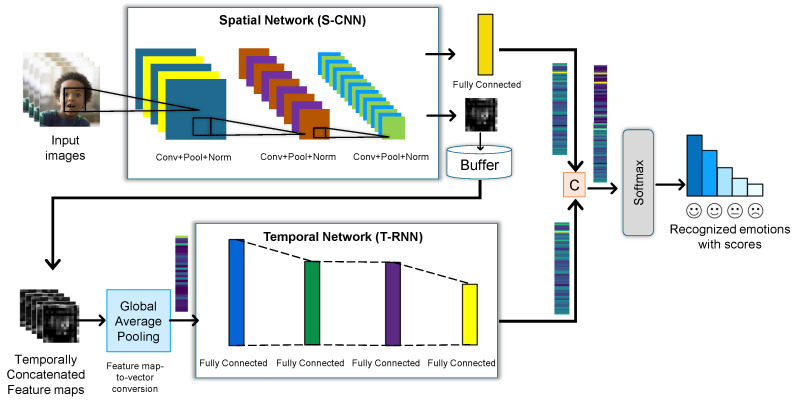
Transfer learning in the proposed FER-Dual-Net Model.

First, a deep neural network model (DNN) is trained over a stream of frames capturing deep facial expression features and classifying them into corresponding emotions. The obtained emotions label vector represents output probability scores for possible facial expressions. We applied both models serially as one of them, the spatial network, for extracting deep features from facial expressions within each frame. However, the other one is a 10-layer network to deal with the temporal features for classifying emotion using frame sequence in a stream to get associated facial emotions. Before passing the extracted 9216-dimensional input features from the fifth pooling layer in the trained CNN network, we first detected face in the frames associated with 500-dimensional vectors. A FER-Dual-Net model is constructed to classify facial emotions using face images as an input supported by transfer learning as demonstrated in Figure 2. It applies changes in training data, hyper-parameters, and ground-truth labels inside the pre-trained models by reducing training time.

#### 3.2.2. Audio-Based Emotion Recognition

**Figure 3:**
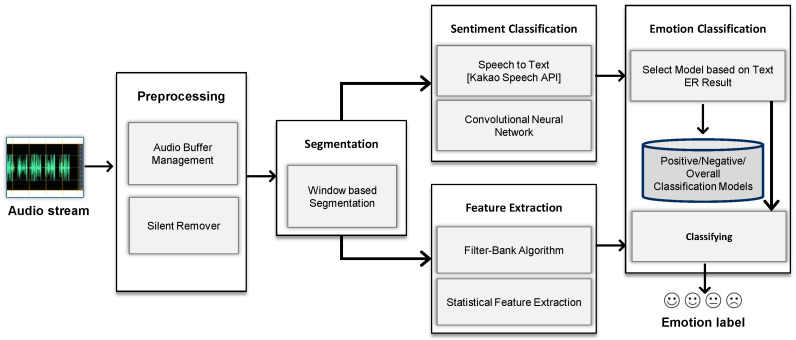
Emotion recognition system based on voice and video.

  Audio features are extracted from speech utterances using the standard speech extraction methods, including fast fourier transform (FFT) and mel-frequency cepstral coefficient (MFCC). These extraction techniques are further reinforced with a method for computing zero crossing rate (ZCR), linear predictive coding (LPC) to extract power spectrum features, spectral centroid, pitch, non-silence ratio, and volume standard deviation to generate audio feature vector [31]. The detailed architecture to recognize emotions from the audio stream is shown in Figure 3. It collects raw audio data streams, removes non-speech area, and performs a 3-sec window-based segmentation using an audio buffer. It further extracted the text from user speech, classified positive/negative information through it, extracted statistical features such as MFCC, LPC, energy, pitch, and finally recognized emotion by using the K-nearest neighbor (KNN) classifier.

Speech text and signal-based emotion recognition systems mainly consist of three components: (1) audio signal pre-processing, (2) speech text-based emotional recognition, and (3) audio signal-based emotional recognition.

Audio signal pre-processing removes unnecessary gaps from audio streaming information collected from the microphone and divides it into variable window sizes suitable for recognition. Speech text-based emotion recognition extracts text from pre-processed data through commercial speech-to-text (STT) APIs and derives positive/negative probability values based on it. Audio signal-based emotion recognition analyzes the signals of voice data processed by the audio pre-processing unit based on the results of voice text-based recognized emotions of the user as a hierarchical structure.

- Audio Signal preprocessing

Audio signals contain patterns of silence in the conversation, which are meaningless and need to be eliminated. The received audio raw signals contain these periods of silence, which are eliminated by observing the frequency content under a threshold value of 15 dB. Again, such a noise is removed as it turns out to be a meaningless speech activity, which is computed using the equation mentioned as under:

(1) $$\mathrm{Ndp}=10log(\frac{P_{r}}{P_{l}})$$

Removal of meaningless non-speech portions such as silence and background noise improves the emotion recognition performance and, ultimately results in an overall increased accuracy.

- Speech Text Extraction

Emotions are also extracted by converting speech into text by using natural language processing (NLP). For this “KoNLPy”, a Hangul (Korean language) commercialized speech emotion recognition Python tool was deployed. It recognizes accurate speech contents by analyzing segmented audio data streams [32].

- Sentiment Classification using CNN

To deal with Korean language text, a morphological analysis was also performed by identifying linguistic units and the structure of morphemes. These include root words, part-of-speech (POS), or affixes. POS tagging ensured marking up morphemes in a phrase using their definitions and contexts. For this, a detailed morpheme analysis and preprocessing data activity were performed using the “KoNLPy” package [33] in addition to Google “Word2vec”. The “KoNLPy” Korean morpheme analyzer was used to preprocess Korean natural language speech data by splitting the target Korean words. The word embedding technique adopted “Word2vec” to map words as data points with similar meanings, which were later deconstructed into morphemes and tokens. These obtained tokens were later vectorized with a value in the embedding layer as input for the CNN classifier, which finally returns emotion class probabilities for text.

- Audio Signal Feature Extraction

The module *Speech Signal-based Emotion Recognition* uses the most common filter-bank implementation to extract feature vectors from raw speech signals windowed over 16 ms [34]. These values comprise of 13 MFCC, 10 LPC, energy, and pitch features per frame. This filter-bank algorithm takes into account human auditory characteristics to extract an overall 52 (13×4) MFCC feature set and is widely used in speech recognition, with proven recognition performance [35]. This filter-bank algorithm is further employed as a speech synthesis method, a widely used approach in speech recognition domain using the human vocalization model. The synthesis process results in the extraction of 40 (10 × 4) LPC features. Lastly, the pitch and energy feature mainly includes the main acoustic correlation of tone and intonation. These are computed through vocal frequency per second, resulting in 4 different features for each of the categories. Thus, a feature vector of length 100 is computed from aforementioned values supported with some additional statistical computations, which mainly included min, max, mean, and standard deviation.

#### 3.2.3. Skeletal-Based Emotion Recognition

**Figure 4:**
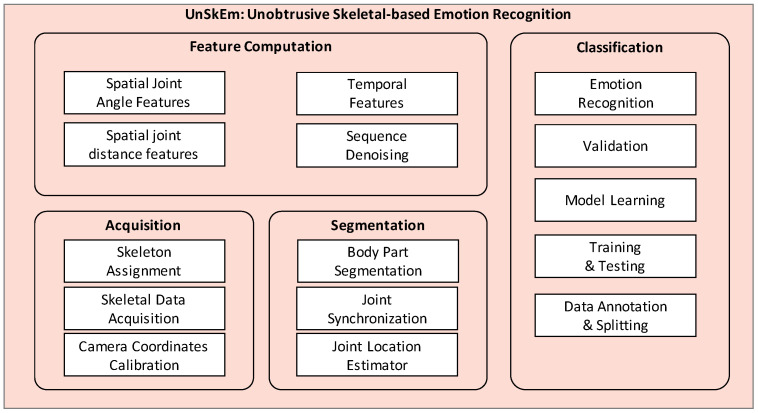
Architecture diagram for an Unobtrusive Skeletal-based Emotion Recognition (UnSkEm) [36].

  We utilized 3D skeleton joint data constituting upper body motion patterns obtained from the Kinect v2 sensor for *Skeletal-based Emotion Recognition*. This utilizes the *Unobtrusive Skeletal-based Emotion Recognition* (UnSkEm) framework for learning emotions through body movements as proposed by Asif et al. [36]. The UnSkEm framework comprises of four sub-modules: *Skeletal Joint Acquisition*, *Skeletal Frame Segmentation*, *Feature Computation*, and *Emotion Classification*, as shown in Figure 4.

Skeletal Joint Acquisition

The skeletal joint information is acquired at the rate of 30 fps (Frames per second) using the Kinect v2 sensor device to track and collect the user’s joints’ 3D coordinates. In this study, we focused only on 15 upper body joints to recognize actions related to body emotions. During the acquisition process, users remained in a sitting posture, with their facial expressions representing specific emotions for approximately 3 min each. The stream representing 15 upper body 3D joint coordinates helped in developing a body language corpus for the UnSkEm framework sufficient to recognize emotions.

Skeletal Frame Segmentation

The acquired arbitrary user actions representing gesture sequences windowed over 3 s further underwent affine transformation. Moreover, these sets of joint’s 3D coordinates are further segmented for feature computation by calculating the angular measurements and inter-joint displacements. During the segmentation process, duplicate 3D joint frames were eliminated to ensure a unique stride in any segment for feature computation.

Feature Computation

**Figure 5:**
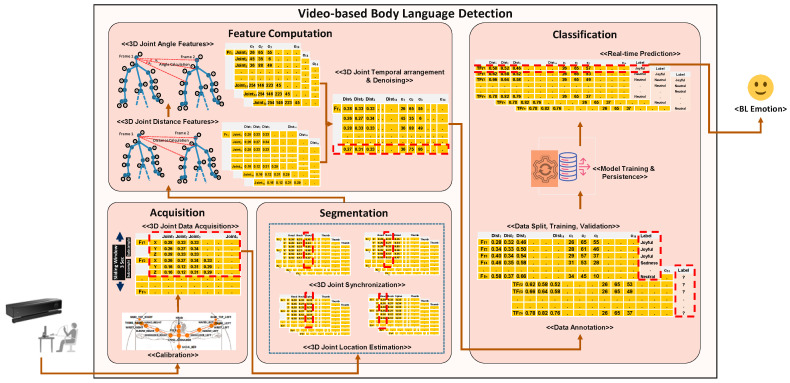
Workflow diagram for an Unobtrusive Skeletal-based Emotion Recognition (UnSkEm).

As mentioned earlier, two different sets of features are extracted from 3D segmented upper body parts: inter-joint displacements and angular measurements. The upper body joints under consideration comprised of the head, neck, hands, elbows, wrists, and shoulders coordinates to analyze behavioral patterns for emotion recognition. Thus, a joint movement for each joint can contribute to a unique emotion. This research work utilizes geometric functions for computing inter-joint distances and angular motion as shown in Figure 5. It also evaluates statistical features like mean, median, and standard deviation. Finally, a feature vector is constructed by involving all neighboring points by using *Mesh Distance Features* (MDF) and *Mesh Angular Features* (MAF) methods. MDF utilizes Euclidean distance measure to extract geometric features (3D joint position and distance), whereas MAF calculates angular features per joint with the rest of the joints under consideration. Thus, by concatenating the results of MDF and MAF, a feature vector of length 280 is obtained and inputted into the deep learning based bidirectional long short-term memory (Bi-LSTM) autoencoder framework [37] as elaborated in Figure 5. The BiLSTM framework, an enhanced version of simple LSTM, has shown its effectiveness when dealing with time-series sequential data [38].

### 3.3. Multimodal Emotion Recognition

In the previous sections, we have discussed *Uni-modal Emotion Recognition* for *Video*, *Audio*, *Text*, and *body language* with independent modalities for emotion recognition. In this section, however, we proposed a framework to address two significant challenges before the fusion actually happened. The challenge of integrating disparate modalities in a fusion method is the synchronization of features computed through variable data in different formats. Creating joint feature vectors that incorporate characteristics from distinct modalities with varying time scales, metric levels, and temporal structures remains an unresolved question for any real-time UX application [39].

**Figure 6:**
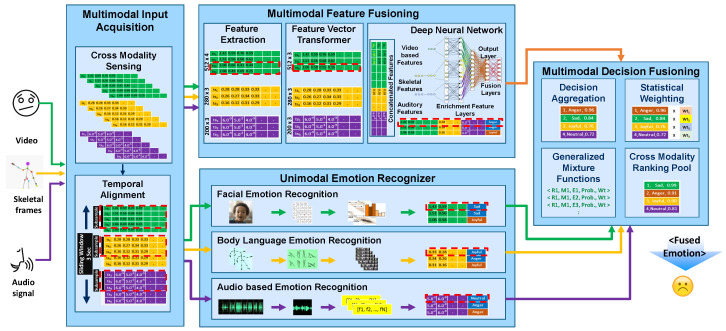
Workflow diagram for an Unobtrusive GM-based Multimodal Emotion Fusion (GM-mmEF).

In the subsequent sections, we discuss solutions to the above-mentioned challenges linked with multimodal feature fusion and decision fusion. These solutions mainly included supervised learning approaches to gather common behavioral patterns linked with a specific emotion. Furthermore, the challenges of accuracy and generalizability associated with multimodal emotion fusion are resolved using the proposed technique for feature fusion and decision fusion as elaborated in Figure 6.

#### 3.3.1. Multimodal Feature Fusion

  The proposed multimodal feature fusion method uses time-synchronized modalities to obtain features and integrates them into a more extended feature vector. Such a technique of integrating features proved to be better for noise handling, as feature-level fusion is subject to low-level information loss. To compensate aforementioned information loss, adaptive systems used classic architectures for fusion by ensuring better noise handling. These architectures mostly depend on Gaussian mixture models (GMM) resulting in reducing the complexity of artificial neural networks (ANN) for enhancing emotion recognition accuracy [40]. In our case, however, we have trained the multi-layer network model using deep learning [41] with features obtained from heterogeneous modalities, concatenated as an input, and produced from a single larger input space. The proposed strategy is illustrated in Algorithm 1.
**Algorithm 1** Multimodal Feature Transformation and Deep Neural Network (DNN) Training**   Input:** $S_{fac}^{FV},\;S_{Aud}^{FV},\;S_{BL}^{FV},\;L_{i}\;\;\;\;\;\;\;\;\;▹$ Unimodal Feature vectors (Video, Audio, Skeletal)
**   Output:** $Emo_{Score}\;\;\;\;\;\;\;\;\;\;\;\;\;\;\;\;\;\;\;\;\;\;▹$ Emotion-Score Vector.
 1:**procedure** MultimodalDeepNetwork 2:   **for all** timestamp t = 1 to T **do** 3:      **function** $FeatureConcatenate(F_{fac},F_{BL},FAud)$ 4:         **if** $(Size\left(F_{fac}\right)=Size\left(F_{BL}\right)=Size\left(F_{Aud}\right)$ **then**        ▹ based on Vector Size 5:            $FV_{FF_{Fac\_Aud\_BL}}\leftarrow F_{fac}∥F_{BL}∥F_{Aud}$ 6:         **else if** $(Size\left(F_{fac}\right)\neq Size\left(F_{BL}\right)\neq Size\left(F_{Aud}\right)$ **then** 7:            $Min_{Idx_{Fac\_Aud\_BL}}\leftarrow Min\left(Size\left(F_{fac}\right),Size\left(F_{BL}\right),Size\left(F_{Aud}\right)\right)$    8:            $F_{fac}\leftarrow Transform\left(F_{fac},Min_{Idx_{Fac\_Aud\_BL}}\right)$ 9:            $F_{Aud}\leftarrow Transform\left(F_{fac},Min_{Idx_{Fac\_Aud\_BL}}\right)$10:            $F_{BL}\leftarrow Transform\left(F_{fac},Min_{Idx_{Fac\_Aud\_BL}}\right)$11:            $FV_{FF_{Fac\_Aud\_BL}}\leftarrow F_{fac}∥F_{Aud}∥F_{BL}$   12:         **end if**13:         Return $FV_{FF_{Fac\_Aud\_BL}}$14:      **end function**15:   **end for**16:   $M_{m\times n}\leftarrow Accumulate\left(FV_{FF_{Fac\_Aud\_BL}}\right)$17:   increment *t* by 3 s18:   $D_{L}\leftarrow M_{m\times n}$19:   **function** $DNNTrainModel(FV_{FF_{Fac\_Aud\_BL}},D_{L},D_{UL},L_{i})\;\;\;\;\;\;\;\;\;\;\;\;\;▹$ input20:      **Forward Propagation**              ▹ initialize training algorithm parameters21:      **Initialize:** Epoch E, Learning rate R, weights W, biases B22:      **Define:** Cost Function C23:      **for** (j=1 to range(epoch)) **do**24:         $D_{F}\leftarrow M_{lab}\;\;\;\;\;\;\;\;\;\;\;\;\;\;\;\;▹$ Retrieve Data (Feature Vectors Matrix)25:         $x_{k}\leftarrow normalize\left(D_{F}\right)\;\;\;\;\;▹$ Pre-process samples, reorder, filter with no missing labels26:         Initialize random weights: ${\left\{w_{1},w_{2},\cdots w_{n}\right\}}^{T}$ and biasness: $\left\{b\right\}$27:         $y=\sigma \left(\sum_{k=1}^{n}w_{k}x_{k}+b\right)\;\;\;▹$ applying nonlinear transformation σ using $y=\sigma \left(w^{T}x+b\right)$28:         $fc_{y}\leftarrow fully\_connected\_NN\left(y\right)$29:         $P_{L_{i}}\leftarrow soft\_max\left(fc_{y}\right)\;\;\;\;\;\;\;\;\;\;\;▹$ Probability distribution for Labels30:      **Backward Propagation**31:         Compute Cross entropy gradient       ▹ Use trained network to predict Emotion labels32:         Apply gradient descent                ▹ Update network parameters33:      **end for**34:      $Emo_{Score}\leftarrow$ Use trained network model                ▹ Predict labels35:      Return *EmotionScoreVector*36:   **end function** 37:**end procedure**


Once the features are extracted from participating unimodal individuals, such as video-based, audio-based, and Kinect v2 sensor, they are concatenated into a more extended feature vector. Thus, a high-dimension concatenated feature vector verily represented rich multimodal data exhibiting the same action simultaneously captured by each sensing modality (i.e., Webcam, Microphone, and Kinect v2 sensor). An important aspect of concatenation, which has to be considered, is balancing variable-sized features so that concatenated features must be of the same length represented by a suitable numerical scale.

The effect of noisy features is reduced by adapting min-max normalization method [42], which proved to have lower error loss and can be calculated using Equation (2) as mentioned below:

(2) $$x^{i}=\frac{x-min(F_{x})}{max\left(F_{x}\right)-min\left(F_{x}\right)},$$

The feature transformer considers three feature spaces $F_{fac},\;F_{BL},\;F_{Aud}$ for facial, body language and audio, respectively. For any arbitrary fused feature sample ϑ, we have $\alpha \in F_{fac},\;\beta \in F_{BL},\;\gamma \in F_{Aud}$. We performed serial feature fusion in which source samples are concatenated into a single global feature, defined as:

(3) $$\vartheta =\left\{\begin{array}{c} \alpha \\ \beta \\ \gamma \end{array}\right\},$$

If the feature vector α is l-dimensional, β is m-dimensional, and γ is n-dimensional then the serially concatenated feature ϑ is (l + m + n)-dimensional serial combined feature space. In case, the dimensions of α,β, and γ are not equal, then the feature vectors with lower dimensionality are padded with zeros until their dimension becomes equal to the others. For explanation, if $\alpha =(f_{1},f_{2},f_{3})$, $\beta =(b_{1},b_{2})$ and $\gamma =(a_{1})$ then the resultant fused feature sample will be $\vartheta =(f_{1}+b_{1}+a_{1},\;f_{2}+b_{2}+0,\;f_{3}+0+0)$. This resulted in video features of the vector size 512, 100-dimensional audio vectors and 280-dimensional skeletal feature vectors, concatenated serially to produce 892-dimensional multimodal feature vector. Since the dimension of the concatenated multimodal feature vector was large enough, subsequently a PCA-based feature reduction technique was used to reduce the dimension of features for improving computation efficiency [43]. It resulted into the reduced 220 multimodal features, which were finally fed into the Deep Neural Network (DNN) learning and classification model.

The DNN consists of an input layer with three hidden layers and a softmax layer to capture the associations between the features from different modalities and classify them into emotions. These hidden units were used by three dense layers activated by *TANH* at a *learning rate* of 0.01, was provided to the softmax classifier layer. The output of the softmax layer represents a vector of size equivalent to the number of emotions with their corresponding probabilities.

#### 3.3.2. Multimodal Decision Fusion

Multimodal decision-level fusion (MDF) aims for a multimodal system towards the effective use of loosely coupled modalities, which makes it more prevalent in numerous fusion techniques.

The extracted multiple feature vectors derived from independent modalities are processed independently by their corresponding classifiers to provide multiple primary emotions with posterior probability score distributions for target emotion recognition. Simple methods exist, such as majority vote, linear as well as non-linear techniques or complex fusion methods for combining independent probability scores to compute the final decision. Such techniques do not have to face challenges offered in the form of noise and failure as they receive pre-processed information.

In this study, we present the use of classifier ensemble methods through weights representing the confidence of individual classifiers. Thus, a traditional fusion method termed ordered weighted averaging functions (OWA) supported by Maximum (Max), Arithmetic mean (Arith), Product (Prod), etc., have been utilized [44].

These aggregation functions are used to combine independent scores through mathematical manipulations associated with dynamic weight selection. Recently, Costa et al. [8] adapted the GM functions, which provide a generalized form of OWA. These functions proved inexpensive and effective with their straightforward utilization in system design and setup, as they offer accurate and robust classifier ensembles. These GM functions use a family of the aforementioned functions instead of a vector of weights for classifier ensembles. The process of fusing emotion information from multiple sources can help to reduce the overall uncertainty in emotion classification, making it more robust and reliable for UX measurement [45].

##### Decision Aggregation

In the decision-level fusion approach, where decision labels and their probability scores, obtained from loosely coupled modalities are further refined. These decisions are further fused in a way to deal with mutual disambiguation amongst heterogeneous modalities to obtain the final emotion decisions.

The most widely used decision-level fusion method avoids synchronization issues as they depend on already processed local decisions. As these local decisions come from individual classifiers dealing with heterogeneous modalities suiting them independently, thus providing them flexibility as compared to other levels of fusions. Several studies exist to aggregate those independent classification score vectors to obtain a single decision having the best score among several classification weight vectors.

To support decision aggregations, we applied mathematical functions to combine the aforementioned multiple classification weight vectors into a single unified output. These aggregation functions transformed *n* emotion attributes with a probability distribution [0,1] interval into a single emotion attribute with the same probability distribution [0,1] interval but a more precise one.

To study and identify challenges associated with multi-criteria decision-making, various aggregation functions have been proposed [46]. These include simpler functions such as arithmetic mean or average, whose results indicate the impact of all representative input vectors. Additionally, extended aggregation functions include geometric mean, harmonic mean, minimum, maximum, product, or bounded sum. A weighted arithmetic mean is used as a common aggregation method wherever group decision-making is required. The class of averaging aggregation functions also includes ordered weighted averaging (OWA) functions in which weights are not associated with particular inputs but with their magnitudes. OWA functions to deal with all sorts of input ranges. For a given weighting vector *w*, $w_{i}\geq 0$, the OWA function is represented as

(4) $${OWA}_{w}\left(x_{1},\cdots ,x_{n}\right)=\sum_{i=1}^{n}w_{i}x_{\left(i\right)},$$

##### Dynamic Statistical Weighting

As discussed previously, emotion labels predicted by the individual classifiers are sent to the combination methods, in addition to a set of weights, obtained from independent modalities, whereas these weights also represented the confidence of the classifiers during the classification process in a combination method. This combination approach also referred to as weighted-based combinations, recommended better composition of an input.

Fundamentally, the combination methods mostly used weights in their functioning; however, in order to apply a weighted-based combination strategy, it is inevitable to define them. So, they are usually defined during the training phase within an ensemble system, whereas they are used throughout the validation or test phase. Such a strategy is called the static weighting process, in which the set of weights is kept constant during the testing or validation phase within the ensemble system. There is a challenge with such a strategy; suppose that an individual classifier dealing with a specific modality received the lowest ranking class score for a particular emotion. So, in a static setting, such classified emotion has a low chance of being considered by an ensemble for the validation and testing phase due to its small static weight, which may lower the overall performance. In order to offer more flexibility and efficiency to an ensemble system, dynamic weight selection can increase performance. For this, GM functions can be used for their unique advantages, as they dynamically utilizes a set of weights for each validation and testing phase. Therefore, GM functions combination method can address the need to define apriori weights for each individual classifier dealing independently with modalities within an ensemble system.

##### Classifier Ensemble Using Generalized Mixture Functions

As mentioned earlier, we applied GM functions as a combination method for a classifier ensemble, supported by dynamic weights, which are determined by each instance of the input vector itself. In this study, we used a multi-view learning approach in which distinct features were assigned to each classifier; however, they all performed the emotion recognition task. Using obtained emotion label score vectors, GM functions are constructed based on a defined referential point for each emotion label vector. The estimated referential point represented a consensus among the opinions of all uni-modal classifiers for each emotion class. They are calculated by any of the GM functions $H_{\theta }$, which are discussed in Algorithm 2. We used $H_{Max}$ (Equation (5)), $H_{Arith}$ (Equation (6)), and $H_{Med}$ (Equation (7)), maximum, arithmetic mean, and median values of output emotion classes, respectively, [8].

(5) $$H_{Max}\left(x_{1},\cdots ,x_{n}\right)=\left\{\begin{array}{cc} x_{1}, & \mathrm{if}\;x_{1}=\cdots =x_{n}\\ \frac{1}{n-1}\sum_{i=1}^{n}\left(x_{i}-\frac{x_{i}\left|x_{i}-Max\left(x_{1}\cdots x_{n}\right)\right|}{\sum_{i=1}^{n}\left|x_{j}-Max\left(x_{1}\cdots x_{n}\right)\right|}\right), & otherwise. \end{array}\right.$$

where $Max\left(x\right)=\underset{i=1\cdots n}{Max}\;x_{i}$,

(6) $$H_{Arith}\left(x_{1},\cdots ,x_{n}\right)=\left\{\begin{array}{cc} x_{1}, & \mathrm{if}\;x_{1}=\cdots =x_{n}\\ \frac{1}{n-1}\sum_{i=1}^{n}\left(x_{i}-\frac{x_{i}\left|x_{i}-Arith\left(x_{1}\cdots x_{n}\right)\right|}{\sum_{i=1}^{n}\left|x_{j}-Arith\left(x_{1}\cdots x_{n}\right)\right|}\right), & otherwise. \end{array}\right.$$

where $Arith\left(x\right)=\frac{1}{n}\sum_{i=1}^{n}x_{i}$,

(7) $$H_{Med}\left(x_{1},\cdots ,x_{n}\right)=\left\{\begin{array}{cc} x_{1}, & \mathrm{if}\;x_{1}=\cdots =x_{n}\\ \frac{1}{n-1}\sum_{i=1}^{n}\left(x_{i}-\frac{x_{i}\left|x_{i}-Med\left(x_{1}\cdots x_{n}\right)\right|}{\sum_{i=1}^{n}\left|x_{j}-Med\left(x_{1}\cdots x_{n}\right)\right|}\right), & otherwise. \end{array}\right.$$

where $\mathrm{Med}\left(x\right)=\left\{\begin{array}{cc} \frac{1}{2}\left(x_{\left(k\right)}+x_{\left(k+1\right)}\right), & \mathrm{if}\;n=2k\;\mathrm{is}\;\mathrm{even}\;\\ x_{\left(k\right)}, & \mathrm{if}\;n=2k-1\;\mathrm{is}\;\mathrm{odd}. \end{array}\right.$

The GM function $H_{\theta }$ is utilized in a two-step process. Initially, the weights of each uni-modal classifier are computed using all the outputs for a specific emotion class. The distance of the uni-modal emotion output is calculated based on the referential point. Further, the ensemble output of each class is calculated, and finally, a maximum value is obtained from the ensemble system for a particular GM-based combination method. The combination method is underpinned by the fusion strategy to fuse the output of individual classifiers with dynamic weights. It considered all individual classifiers irrespective of their uni-modal emotion weight vectors in terms of posterior probabilities.

##### Cross-Modality Ranking Pool

GM mixture functions preserve the cross-modality emotion ranking and provide a combination method, as discussed earlier. However, it enhances any uni-modal emotions by combining them with dynamic weights.

We used the GM mixture model over the emotions maintained in *Cross Modality Ranking Pool*. GM functions ensured the interaction among various modalities by reducing individual modality probability variations in emotion score vectors and providing solid generalizability within modality-modality variability. The proposed methodology ensured and maintained a higher-order relevancy amongst multimodal entities. This also ensured emotion discriminability by correlating and exploiting the relations among emotions obtained from heterogeneous modalities.
**Algorithm 2** Multimodal Decision Level Fusioning (GM-based Combination method $H_{\theta }$)   **Input:** Dataset D of size N with instances $I_{N}$ for modalities $M_{j}$ classifying emotions $E_{k}$ with posterior probabilities $P_{jk}$.   **Output:** $E_{dec}\;\;\;\;\;\;\;\;\;\;\;\;\;\;\;\;\;\;\;\;\;\;\;\;\;\;\;\;▹$ Highest ensemble score.
 1:**procedure** Multimodal Decision Fusion                        ▹ Gets Input Matrix 2:   $V_{j}^{N}=\left\{P_{j1}^{N},P_{j2}^{N}\cdots ,P_{jk}^{N}\right\}\;\;\;\;\;\;\;\;\;\;\;\;\;▹$ vectors representing all emotions in a modality. 3:   **for** i = 1 to N**do**                              ▹ For each instant in D 4:      **for** i = 1 to k **do**                              ▹ For each $E_{k}$ for $M_{j}$ 5:         $R_{\theta ,k}^{N}\leftarrow$ Compute Referential Point for each $E_{k}$            ▹ θ in {Max,Arith,Prod⋯} 6:         $d_{k}^{N}\leftarrow \sum_{i=1}^{N}\left|P_{jk}^{N}-R_{\theta ,k}^{N}\right|\;\;\;\;\;\;\;\;\;\;\;\;\;\;\;\;\;\;\;\;\;▹$ Compute Sum of Distances 7:      **end for** 8:      **while** i≤k **do**                        ▹ Compute weight for each emotion $E_{k}$ 9:         **if** $d_{k}^{N}>0$ **then**10:            Weight calculation11:            $w_{k}\leftarrow \frac{1}{k-1}\left(1-\left(\frac{\left|P_{jk}^{N}-R_{\theta ,k}^{N}\right|}{d_{k}^{N}}\right)\right)\;\;\;\;\;\;\;\;\;\;\;\;\;\;\;▹$ Weight of each modality w.r.t. $E_{k}$12:         **else**13:            $w_{k}\leftarrow \frac{1}{k}$14:         **end if**15:         **return** $w_{k}\;\;\;\;\;\;\;\;\;\;\;\;\;\;\;\;\;\;\;\;\;\;\;\;\;\;\;▹$ The Weight vector.16:      **end while**17:      $O_{k}^{ens}\leftarrow \sum_{j=k=1}^{N}\left(P_{jk}^{N}\cdot w_{k}\right)\;\;\;\;\;\;\;\;\;\;\;\;\;\;\;▹$ Compute ensemble output for each $E_{k}$18:      $E_{dec}\leftarrow Idx_{Max}\left(O_{k}^{ens}\right)$19:   **end for**20:**end procedure**


## 4. Experimental Evaluations

This section presents the implementation methods to evaluate the proposed framework using three experiments performed to determine accurate human emotions. These experiments included unimodal methods for individual modalities, multimodal feature-level fusion, and GM function-based decision-level fusion. To evaluate these methods, we used confusion matrices for each emotion. The fused emotion accuracies and decision fusion emotion scores supported each modality’s through detailed analysis. Moreover, the section also presents the datasets, framework validation using suitable evaluation metrics, and finally, comparisons are drawn with state-of-the-art.

### 4.1. Dataset and Implementation

The recognition process involved 4 candidate emotions *Happiness*, *Neutral*, *Sadness*, and *Anger*, portrayed by 10 participants with ages between 22 and 35 years. They included university-enrolled students, equal in gender (five male and five female) of mixed race to evaluate the Lean UX Platform [25]. All experiments were performed in a controlled lighting environment, with each participant guided about different frontal face positions and upper body movements in front of the webcam at a minimum distance of 1.5 m. These users were allowed to move and react freely at a maximum distance of 4 m. The dataset was collected from different modalities by deploying devices such as Kinect v2, webcam, and microphone within the sessions of approximately 15 min for each participant. A special desktop application under the LeanUX framework was developed to collect data. The proposed H-MMER framework was deployed on a computer running the Windows 10 OS, and is equipped with an Intel i-7 processor, 16 GB of RAM, and a 6 GB graphics card.

The dataset pool for detecting body language and face images consist of a total of 216,000 frames captured at a rate of 30 fps from webcam and Kinect v2. These frames were collected from 10 users performing each emotion for approximately 3 min according to list of actions. To elaborate, there are 55,300 frames that have been categorized as expressing *Happiness*, 55,700 frames that depict a *Neutral* emotion, 54,240 frames that display *Sadness*, and 50,760 frames that exhibit *Anger*. The framework is designed to update the emotion result each 3 s, i.e., features are extracted from each 90 frames to be classified into an emotion label.

Each body language frame comprises 45 parameters, which include the x, y, and z coordinates of 15 skeleton joints that are used to represent one of the four emotions. It is important to observe that in each frame, all 15 3D points are detected completely without any overlaps or missing points. After preprocessing we extracted around 280 features using *MDF* and *MAF* methods. Similarly, webcam collected a stream of frames of size 1024×720 at a rate of 30 fps for human face detection and facial emotion recognition. In total, we obtained 512 *HoG* and *SIFT* features for ROIs in a frame for detecting face.

Additionally, the voice samples were also recorded using a microphone by each of the participants who were instructed to speak approximately 40 pre-scripted sentences in the Korean language, 10 for each emotion. These sentences were spoken at varying levels of intensity (high, medium, and low) and speech rate. Around 2750 voice samples were finalized by the LeanUX platform’s expert team, who categorized them into different emotions. Specifically, there were approximately 620 voice samples for *Happiness*, 830 for *Neutral*, 760 for *Sadness*, and 540 for *Anger*. After undergoing preprocessing, approximately 100 features were computed for audio emotion recognition. These features consisted of 52 MFCC, 4 LPC, 4 Energy, and 4 Pitch, each evaluated for 4 statistical measures such as standard deviation, mean, minimum, and maximum with detailed discussion in Section 3.2.2.

We pre-processed the data and independently extracted different features depending on the modality. We obtained video feature vectors of dimensions m × n, skeletal features p × q, and audio features s × t, where m, p, s represent number of features and n, q, t represents number of instances, respectively. These feature vectors are fed to individual classifiers for emotion recognition, whereas for a multimodal feature and decision fusion, these feature vectors undergo transformation by keeping n, q, and t equal to a feature vector size based on the lowest estimated size. The equal-sized feature vectors are then concatenated to perform multimodal feature and decision fusion.

### 4.2. Multimodal Emotion Recognition Results

In our comprehensive evaluation discussions, we described multiple evaluation tasks, firstly for each modality and then for a multimodal feature and decision fusion. We reported confusion matrices and accuracies for predicted emotion categories.

#### 4.2.1. Performance Analysis of Video-Based Emotion Recognition

In order to recognize video-based emotions, the developed component extracted feature from an input image and recognize emotion using multi-class logistic regression (softmax) classifier. The softmax classifier uses information theory-based ranking criteria to calculate probabilistic emotion scores. These scores represented multiple outputs with specific confidence for each predicted emotion label. So the output of the FER-Dual-Net model interpreted the user’s facial emotions more objectively using a multi-class logistic regression classifier. The confusion matrix for video-based emotions is shown in Table 1. It is found that among the accuracies of four emotion labels, the *Happiness* and *Anger* classes get higher accuracies of 95% and 94.95%, respectively. The classifier well-recognized these emotions due to the discriminative facial characteristics within the spatio-temporal domain. *Neutral* and *Sadness* emotions, however, have lower accuracies of 89.13% and 86.75%, respectively. These two emotions are indistinguishable due to normal facial expressions, eyebrow motions, landmarks, and wrinkles around the nose region or head pose.

To demonstrate the effectiveness of the proposed approach, partial AffectNet [47] dataset is utilized for the evaluation by comparing mean emotion accuracy with the state-of-the-arts. We utilized a subset of facial expression images in AffectNet, a large database of facial expressions, arousal, and valence in the wild that allows for automated facial expression recognition. We used 4 basic emotion labels for evaluation, which includes *Happiness*, *Neutral*, *Sadness* and *Anger*. For each emotion category, we randomly selected 1000 images (80% for training and validation while the remaining 20% for testing) to train the proposed facial expression recognition model. Furthermore, the benchmark dataset was evaluated and the proposed model achieved a total accuracy of 91.46%, surpassing the state-of-the-art models listed, including Gan et al. [48] with 88.05% and Hua et al. [49] with 87.27% as shown in Table 2. It can be implied that the coordination between spatial feature maps and temporal feature vectors is facilitated by the effective capture of underlying properties of facial emotions in the spatio-temporal domain.

#### 4.2.2. Performance Analysis of Audio-Based Emotion Recognition

We reported the classification performance of audio emotions for the extracted features as described in Section 3.2.2. Audio emotions are recognized using the two-fold method, first, speech-text emotions are recognized over the segmented 3-s audio stream. Secondly, Speech signal-based emotions are recognized over the segmented 3-s audio stream and scores are obtained for speech-text emotions. The text sentiment was recognized using Text-CNN in Tensorflow evaluated by 4-fold cross-validation, whereas the final Audio Signal based emotion was recognized using KNN supported by WEKA API with 10-Fold cross-validation over 80% training and 20% test data. The KNN classifier model utilizes speech-text emotion-based scores as a basic heuristic rule.

Table 3 shows the confusion matrix of speech signal-based emotion recognition with an accuracy of 66.07%. According to the findings, the emotion *Anger* received the highest recognition rate of 71.5%, while *Happiness* was recognized with a lower accuracy of 68.2%. Among all the emotions, *Sadness* had the lowest accuracy, measuring at 58.7%.

The findings in bold as shown in Table 4 indicate that deep learning based hierarchical structure proposed by Singh et al. [50] has the potential to recognize emotions from speech with greater accuracy than our proposed method using simpler algorithm. However, our proposed approach for audio-based emotion recognition requires low computational resources since it utilizes a dataset with 4 emotions that is less complex, making it suitable for real-time LeanUX evaluation.

#### 4.2.3. Performance Analysis of Skeletal-Based Emotion Recognition

In order to prove the proposed methodology, the experiments were carried out to correctly classify skeletal-based emotions using skeletal joint sequences of similar actions. The BiLSTM framework was applied to classify these multi-class labels for emotions. We can consider the human skeleton as series of interconnected of joints, where the motion and position of one joint may impact the others in a specific order. In our case, BiLSTM framework utilizes skeletal join data to train network for evaluating the body language emotions required for the LeanUX Platform. BiLSTM scaled well with the variable sizes of training data and proved efficient experimentally. We divided joint, skeletal data into two halves, i.e., 80% for training and validation while the remaining 20% for testing. The proposed methods, MDF and MAF, were utilized to extract features concatenated linearly to represent similar emotions. A 3-layer BiLSTM implemented in PyTorch was employed to train the softmax classifier, using the Adam optimizer with an initial learning rate of 0.01. The training process utilized a dropout rate of 0.1 and was carried out over 5 epochs with a split size of 5.

The detailed accuracy analysis is presented in Table 5, which shows an accuracy reaching 97.01%. The *Happiness*, *Neutral*, and *Sadness* emotion had better accuracies, which were 97.71%, 98.67%, and 96.22%, respectively. The *Anger* emotion, however, could achieve 95.42% lower accuracy.

The results presented in Table 6 demonstrate the effectiveness of the deep learning BiLSTM framework in the proposed method for the recognition of emotions using the LeanUX dataset. The mean recognition accuracy obtained through this approach is higher mentioned in bold to that achieved by other methods employed for skeletal-based emotion recognition.

#### 4.2.4. Performance Analysis of Multimodal Emotion Feature Fusion

The implementation of the *Multimodal Feature Fusion* for emotion recognition was done using *Deep Neural Network* supported by an open-source, distributed deep-learning library *Deeplearning4j*, written for Java and other languages [41]. After extracting features from different modalities, we used feature fusion methods to combine high-level features from different modalities into a long feature vector to form a joint feature representation.

After receiving the multimodal features from facial, skeletal, and audio modalities, the size of the feature vector was determined in terms of length. We received features of order mxn where m represents a number of features and n represents a number of tuples in a sliding window of 3 s. For equal-sized feature vectors, concatenation was performed. Whereas for variable sizes, selected the n-tuples of feature vectors were using a threshold set by the modality having the lowest tuple size from the buffer within the sliding window. So, concatenation was performed based on the lowest estimated number of tuples. The resulting feature vector as discussed in Section 3.3.1 is set for an input into the DNN algorithm for training to classify 4 emotions.

In order to train the DNN model, we used 3-layered architecture, with the first two fully connected dense layers and the third one for output, together with back-propagation to adjust the entire framework. We set the batch size to 1500 over the training dataset consisting of epochs. The aforementioned dense layers employed the *Xavier* weight initialization method in addition to the *TANH* activation function for the convergence over the normalized feature vectors with a learning rate of 0.01. These hyper-parameters were used in batches over the initial number of training rows to classify emotions using the *softmax* classifier with a negative log-likelihood loss function. The multimodal emotion feature fusion output is a corresponding emotion vector coming from different modalities.

The confusion matrix in Table 7 shows higher accuracies for emotions of *Happiness*, *Neutral*, and *Sadness* with values of 98.21%, 98.85%, and 98.08%, respectively, as they were identified by the model well. Meanwhile, the Anger emotion received an accuracy of 95.68% in the multimodal feature fusion lag behind slightly.

A comparison of accuracies is reproduced in Table 8 for different experiments performed involving several modalities. The results suggest a higher accuracy is achieved for the multimodal feature fusion method.

#### 4.2.5. Performance Analysis of Multimodal Decision Level Fusion with GM Functions

Multimodal decision-level fusion does not require feature vectors as the multimodal feature fusion method. Instead, obtaining emotion feature vectors from different modalities with an individual classification probability value requires another merging technique. In our experiments, we used dynamic weighting as a combinational input method for merging the probabilities of each emotion vector obtained from individual modalities and then selected the emotion label with the highest computed score.

In order to investigate and illustrate the feasibility of the proposed approach as a combination module of an ensemble system, an empirical analysis is also conducted. In this analysis, the obtained ensembles are applied to multimodal datasets gathered for the LeanUX project as described in Section 4.1. As described, each dataset’s number of instances, classes, and attributes were applied for multimodal decision fusion using GM functions [8].

Three-membered ensembles were composed of three classification algorithms an individual classifier and a multimodal feature fusion output. Thus, the proposed framework for multimodal decision fusion evaluated unimodal, multimodal feature fusion and compares it with multimodal decision fusion methods using functions such as Maximum (Max), Product (Prod), Arithmetic mean (Arith), and Product (Prod). It further utilized a re-sampling procedure similar to bagging, so a change in the parameter setting of an individual classifier for the aforementioned individual classifier for ensembles is not thus required. The combination module, however, used the following GM functions $H_{Max}$, $H_{Prod}$, and $H_{Arith}$.

In our experiments, we analyzed that GM functions achieved better statistical significance over a single combination method or on an individual classifier. The study conducted by Costa et al. [8] proved $H_{Max}$ and $H_{Arith}$ to be the best performers, with the GM functions combination method having a smaller number of ensembles. Furthermore, an increase in the number of classifiers had a positive effect on the performance of combination methods, $H_{Max}$, $H_{Prod}$, and $H_{Arith}$. On the other hand, an increase in the number of classifiers negatively affected the performance of classical combination methods.

A detailed analysis using different columns in Table 9 suggests improved and stable accuracies for bimodal, trimodal, and multimodal decision fusion using GM functions. For $H_{Max}$ and Prod with ensemble sizes 2, 3, and 4 provided higher accuracies. An interesting aspect is that the classical combination method, such as *Prod* proved to be a better performer in lower ensemble sizes as we have limited modalities, so there is less variation in the ensemble size. For this reason, uniform patterns in the accuracies can be observed using classical combination methods and GM functions.

Finally, an analysis is performed over the results obtained from multimodal decision fusion as depicted in the confusion matrix shown in Table 10. The matrix indicates a higher accuracy for each emotion without abrupt accuracy changes. Our proposed approach achieved an overall accuracy of 98.19% using the GM function combination method. The higher accuracy indicates the efficacy of the dynamic combination method for the emotion recognition process.

### 4.3. Comparison of Unimodal, Multimodal Feature Fusion and Decision Level Fusion Results

**Figure 7:**
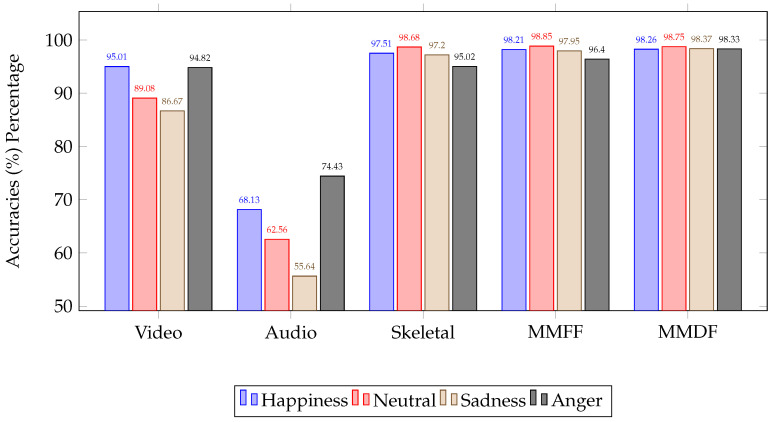
Unimodal, multimodal feature fusion and decision level fusion.

The accuracy for all of the obtained emotions using different experiments, as described in the earlier sections, is summarized in the plot shown in Figure 7. This plot shows accuracies of individual emotions for unimodal classification, combined accuracies for multimodal feature fusion, and final emotion results obtained from multimodal decision fusion. The graphical representation proved multimodal decision fusion as the most significant among the other individual models and fusion methods.

This research provides a detailed investigation and analysis of the combination method using dynamic weight selection in variable ensembles based on GM functions. The final decision is built on combination method of each modality prediction using GM functions $H_{Max}$, $H_{Prod}$, and $H_{Arith}$. High-performance results are achieved using multiple levels of fusion. These results demonstrated that GM function-based multimodal decision fusion outperformed unimodal and multimodal feature fusion for human emotion recognition *accuracy*. The high performance of the proposed methodology resulted in dynamic weight selection through an ensemble technique using GM functions for multimodal decision-based emotion fusion. Furthermore, the performance results obtained demonstrated that the combination method also provided a generalization of emotion fusion performance for better classification using multimodal decision fusion. As demonstrated, the proposed method has been proven for promising results in developing a comprehensive emotion fusion framework in comparison with state-of-the-art studies as shown in Table 11.

## 5. Conclusions and Future Work

In this paper, we studied challenges associated with multimodal fusion, one of the main research issues on multimodal emotion recognition. We introduced the Hybrid Multimodal Emotion Recognition (H-MMER) framework, which fuses features at the decision level, the multimodal feature level, and the unimodal feature level. This research added two significant new contributions to an earlier multimodal fusion work on emotional recognition. To begin with, we considered the input modalities (sensors) as sources of rich temporal event streams that included important multimodal data. Therefore, to gather multimodal data as user session logs required for defining the appropriate UX metrics, we developed a multimodal input collection module that allowed cross-modality sensing (CMS) and conducted temporal alignment (TA) of stream events. Second, we specified several fusion modes and levels to increase fusion’s accuracy. We presented a novel approach using Generalized Mixture (GM) functions in User Experience (UX) domain. These GM functions included combination methods $H_{Max}$; $H_{Arith}$ and $H_{Med}$ to perform decision-level fusion in classifier ensembles. In the analysis, we compared the proposed approach with unimodal, bimodal, traditional combination methods, Maximum (Max), Arithmetic mean (Arith), Majority vote (Vote), and Product (Prod). We have included the suggested framework in an ongoing research platform known as the "LeanUX platform" for modeling UX metrics based on multimodal data for emotional UX evaluation. The empirical analysis suggested that the generalized mixture functions $H_{Max}$; $H_{Arith}$, and $H_{Med}$ can be used as a combination method to design an accurate classifier ensemble. The experiment demonstrates that the suggested framework has an average accuracy of 98.19% in simulating emotional states. Overall assessment results demonstrate our ability to precisely identify emotional states and improve an emotion recognition system required for UX measurement.

## Figures and Tables

**Table 1:** Confusion Matrix for video-based emotion recognition.

Mean Classification Acccuracy: (91.46%) & Classification Error: (8.54%)					
Type of Emotions		Emotion Recognition Rate (%)			
		Happiness	Neutral	Sadness	Anger
Ground Truth	Happiness	95.0	2.01	1.40	1.60
	Neutral	0.01	89.13	10.90	0.02
	Sadness	4.61	4.13	86.75	4.60
	Anger	0.40	4.67	0.12	94.95

**Table 2:** Comparison results of video-based emotion recognition with the state-of-the-art.

State-of-the-Art methods	Datasets	Number of Emotions	Mean Recognition Accuracy (%)
Gan et al. [48] *	*AffectNet* [47]	4	88.05
Hua et al. [49] *		4	87.27
**Proposed Video-based ER**	*LeanUX* [25]	4	**91.46**

* The results are realized by our own implementation and for a fair comparison, the default configurations for algorithms as proposed in their respective research papers are used.

**Table 3:** Confusion Matrix using trained KNN for audio-based emotion recognition.

Mean Classification Accuracy: (66.07%) & Classification Error: (33.93%)					
Type of Emotions		Emotion Recognition Rate (%)			
		Happiness	Neutral	Sadness	Anger
Ground Truth	Happiness	68.2	4.7	8.4	18.8
	Neutral	13.5	62.5	17.7	6.2
	Sadness	17.3	21.01	58.7	3.1
	Anger	1.99	11.8	14.8	71.5

**Table 4:** Comparison results of audio-based emotion recognition with the state-of-the-art.

State-of-the-Art Methods	Datasets	Number of Emotions	Mean Recognition Accuracy (%)
Deb et al. [51]	IEMOCAP [52]	6	66.80
Singh et al. [50]	RAVDESS [53]	8	**81.20**
**Proposed Audio-based ER**	LeanUX [25]	4	66.07

**Table 5:** Confusion Matrix for Skeletal-based emotion recognition.

Mean Classification Acccuracy: (97.01%) & Classification Error: (2.99%)					
Type of Emotions		Emotion Recognition Rate (%)			
		Happiness	Neutral	Sadness	Anger
Ground Truth	Happiness	97.71	0.18	0.41	1.71
	Neutral	0.12	98.67	0.34	0.85
	Sadness	0.29	0.44	96.22	1.50
	Anger	1.12	0.54	2.80	95.42

**Table 6:** Comparison results of Skeletal-based emotion recognition with the state-of-the-art.

State-of-the-Art Methods	Datasets	Number of Emotions	Mean Recognition Accuracy (%)
Razzaq et al. [36]	UnSkEm [36]	6	96.73
Shi et al. [54]	Emilya [52]	8	95.50
**Proposed Skeletal-based ER**	LeanUX [25]	4	**97.01**

**Table 7:** Confusion Matrix using multimodal emotion feature fusioning.

Mean Classification Acccuracy: (97.71%) & Classification Error: (2.29%)					
Type of Emotions		Emotion Recognition Rate (%)			
		Happiness	Neutral	Sadness	Anger
Ground Truth	Happiness	98.21	0.10	0.27	1.42
	Neutral	0.21	98.85	0.10	0.84
	Sadness	0.10	0.04	98.08	1.91
	Anger	1.46	1.01	1.10	95.68

**Table 8:** Comparative results: Feature Fusion Comparison Table.

Modality	Accuracy (%)
Video-based ER	91.46%
Skeletal-based ER	97.01%
Audio-based ER	66.07%
**Multimodal Feature Fusion**	**97.71**%

**Table 9:** Results: Accuracies for ensembles using *GM* combination methods.

Modality	EnsSize	Vote	Max	Arith	Prod	*H_Max_*	*H_Arith_*	*H_Prod_*	Best
Aud_Fac	2	0.808	0.808	0.800	0.783	0.841	0.808	0.799	*H_Max_*
Aud_BL	2	0.810	0.810	0.808	0.810	0.824	0.816	0.824	*H_Max_* & *H_Prod_*
Fac_BL	2	0.941	0.941	0.941	0.994	0.943	0.944	0.944	Prod
Aud_Fac_BL	3	0.943	0.943	0.943	0.949	0.941	0.949	0.942	Prod & *H_Arith_*
Aud_Fac_BL_FF	4	0.978	0.978	0.979	0.972	0.982	0.972	0.974	*H_Max_*

**Table 10:** Confusion Matrix: Multimodal Decision Fusion using GM function.

Mean Classification Acccuracy: (98.19%) & Classification Error: (1.81%)					
Type of Emotions		Emotion Recognition Rate (%)			
		Happiness	Neutral	Sadness	Anger
Ground Truth	Happiness	98.47	0.11	0.14	1.49
	Neutral	0.06	98.79	0.12	1.07
	Sadness	0.98	0.44	98.49	0.21
	Anger	0.11	0.61	0.93	97.01

**Table 11:** Comparison results of the proposed *H-MMER* framework with state-of-the-art Multimodal Emotion Recognition methods.

State-of-the-Art Methods	Datasets	Number of Emotions	Mean Recognition Accuracy (%)
Middya et al. [2]	RAVDESS [53]	8	86.00
	SAVEE [2]	8	**99.00**
Hussain et al. [9]	LeanUX [25]	7	95.80
**Proposed H-MMER**		4	**98.19**

## Data Availability

Due to project work and restrictions data will be shared later otherwise code is available at Github.

## Abbreviations

The following abbreviations are used in this manuscript:
UX	User Experience
  HCI	Human–Computer Interaction
  FC	Feature Concatenation
  FER	Facial Expression Recognition
  LPC	Linear Predictive Coding
  OWA	Ordered Weighted Averaging
  GMF	Generalized Mixture Functions
  GM-mmEF	GM-based Multimodal Emotion Fusion
